# Development of a bio-inspired wound model for debridement training

**DOI:** 10.1007/s44164-024-00071-6

**Published:** 2024-04-29

**Authors:** Jessica J. Senior, Kayley Jaworska, Leanne Atkin, Karen Ousey, Alan M. Smith

**Affiliations:** 1 Department of Pharmacy, University of Huddersfield, Queensgate, Huddersfield, HD1 3DH UK; 2 Institute of Skin Integrity and Infection Prevention, University of Huddersfield, Queensgate, Huddersfield, HD1 3DH UK; 3 Mid Yorkshire NHS Teaching Trust, Wakefield, UK

**Keywords:** Wound model, Debridement, Slough, Eschar, Biopolymer, Simulation training

## Abstract

Wound debridement is commonplace in expediting wound healing in the clinic. Despite this, there are limited resources available for simulation training for practitioners prior to facing real-life patients. Typically, citrus peels or porcine skin are employed in a vain attempt to improve debridement proficiency, yet these fail to provide a realistic experience of the textures and consistencies of wounds. Therefore, there is a clear unmet need for a safe and effective tool that can facilitate hands-on learning under the instruction of an experienced debrider. To fill this niche, a life-like wound model was designed and developed, featuring leathery necrotic eschar, an intermediary sloughy layer, and a rough granulation tissue in the bottommost layer, all within a healthy skin base. The healthy tissue portion of the model was designed to exhibit similar mechanical properties to those found in human skin. Likewise, the sloughy layer was viscous enough to remain within the model under static conditions yet could be removed using any appropriate debriding tool synonymous with real slough. Mechanical testing of the necrotic eschar revealed brittle fracture behaviour, akin to what is observed in patients. Each layer of the wound model provided the visual and haptic feedback of how it would look and feel to debride a patient’s wound in the clinic, giving invaluable experience for potential trainees in a safe and effectual way. It is envisaged that these models can be developed in a personalised way to suit the individual needs of the user, such as incorporating underlying models of bone or tendon, while retaining the key elements of the wound, which make it successful. This model is proposed as an important step forward in bridging the gap between becoming a newly qualified debriding practitioner and encountering the first wound in the clinic, subsequently improving the confidence of the debrider and enhancing patient outcomes.

**Supplementary Information:**

The online version contains supplementary material available at 10.1007/s44164-024-00071-6.

## Introduction

Wound debridement is the act of removing devitalised tissue and continues to be a cornerstone of wound management [1]. Debridement involves the careful removal of impurities such as nonviable tissue (including slough and necrotic eschar), bacterial contamination, scab formation, and other obstacles present in the wound bed that contribute to delayed wound healing [2]. There are many different methods of wound debridement, grouped into autolytic, surgical/sharp, mechanical, and biological categories, and each has their associated advantages and disadvantages. Appropriate and early debridement accelerates wound healing, which in turn delivers additional benefits of improved quality of care, enhanced patient health and wellbeing, and a reduction in treatment costs [3]. Among these approaches, mechanical and sharp debridement is considered the speediest methods of wound debridement.

Despite the upmost importance of performing wound debridement efficiently, existing wound models used for debridement training and education suffer from drawbacks such as high costs and an inability to realistically replicate the appearance and textures of human tissue and real-life wounds. Commonly employed methods involve utilising citrus fruits, including orange or grapefruit peel [4], or pig’s feet [5], but these approaches have notable limitations. While citrus peel may bear some resemblance to the texture of specific necrotic wound tissues, it inadequately simulates the intricate biological and physiological properties associated with various wound types encountered by clinicians. Consequently, using inappropriate materials for wound debridement instruction can result in disseminating incorrect information and potentially jeopardise patient safety. Although porcine tissue offers greater anatomical realism, its use raises ethical concerns surrounding the utilisation of animal tissue for teaching purposes, along with concerns about infection risk, limited representation of wound types, lack of realism, and difficulties in sourcing, particularly in remote regions when procuring large quantities or shipping poses challenges [6].

The ability of clinical staff to become competent in mechanical debridement is difficult, and many nurses frequently struggle to access the appropriate education and training required [7]. This is due to a number of factors, including limited access to appropriate wound models or simulation education, leading to the only method of education being within clinical settings of ‘see one – do one’—a concept of education which is becoming less acceptable due to concerns over patient safety [8]. Synthetic training models may be utilised during educational workshops to visually resemble real wounds; however, they lack the intended purpose of being training devices as they are designed solely for observational purposes. The only interactive model known in the field employs a combination of polystyrene foam, clay, paint, leather, and glue to create a reusable model [9]; however, despite being a useful step forward, none of these materials exhibits bulk mechanical properties comparable to actual tissue.

Although wounds are personalised due to individual aetiology and are biologically complex, there are common physical features that clinicians are faced with when debriding chronic wounds. Generally, there is a wound void with rough granulation tissue lining the wound bed, above which there can often be a layer of yellow or white sloughy tissue, which may be loosely or firmly adhered [10, 11]. In some severe chronic wounds, there is often a ‘leathery’ necrotic eschar that requires sharp debridement, which, without proper removal, can act as a source of nutrition for bacterial contaminants [2, 12]. It should also be noted that healthy skin usually surrounds chronic wounds and all these different tissues within and surrounding the wound area have very different mechanical behaviours. In healthy skin, the extracellular matrix (ECM) is composed of a complex network of biopolymers such as proteoglycans, which are long-chain polysaccharides, and fibrous proteins such as collagen. These materials influence the mechanical properties of the skin due to their ability to retain water and form hydrated gels, contributing to the skin’s hydration, elasticity, viscoelastic behaviour, and overall mechanical integrity [13]. Within wounded tissues, sloughy tissue also exhibits viscoelastic behaviours due to the high moisture content and presence of exudate within the tissue, allowing the material to flow and deform under external forces such as sharp debridement [14]. Moreover, the ECM within sloughy tissue often undergoes changes due to the presence of non-viable tissue, and these disruptions to the ECM’s structural components can contribute to reduced elasticity and increased viscosity [15]. This can impact the ability to remove the slough effectively through debridement techniques, as it may adhere to the wound bed or have a higher resistance to mechanical forces. The necrotic eschar consists of non-viable, necrotic tissue, often composed of coagulated blood, fibrin, and denatured proteins [16]. ECM components such as collagen and glycosaminoglycans can also be present in eschar. However, these tend to be dehydrated due to the lack of moisture, contributing to its hard, leathery texture [14, 17].

The aim of this work, therefore, was to develop an enhanced and anatomically realistic wound model using biopolymer materials that more realistically replicate chronic wounds as they manifest in clinical settings. These models could be subsequently used as a tool for trainee clinicians to safely practice sharp debridement.

## Methods

A variety of methods were used to produce initial prototypes, which included preparing 3D printed moulds, preparation and characterisation of the biopolymer materials, and building and integrating the wound layers.

### 3D printed mould design

Based on clinical evaluations of wounds observed in a vascular clinic, a chronic wound mould of sufficient diameter and depth and with characteristic features was designed. Initially, the wound mould base and surrounding casing were designed using Meshmixer in the shape of a cuboid so that the dimensions of the final casted model would equate to H 8.5 cm × W 12.5 cm × D 2.0 cm. The mould base was designed to feature smooth, healthy skin, within which the wound void was situated. The dimensions of the wound were H 6.5 cm × W 8.5 cm × D 1.0 cm and featured rough granulation tissue in the centre and a callous edge at the perimeter—akin to the physiological features of real-life wounds. The wound mould base was also reverse-engineered to produce a mould to facilitate the fabrication of surface eschar that was complementary to the dimensions of the wound void. Both mould designs were saved as STL files in preparation for setting pre-print parameters. Mould files that were printed using a Creality Ender-3 Pro 3D printer were modified using Creality Slicer software, where a suitable print path was computed as well as tailoring print resolution. Mould files that were printed using a Prusa MK3S + 3D printer were modified using PrusaSlicer and rendered into GCODE as the readable print file prior to printing. All moulds were printed using polylactic acid (PLA) thermoplastic.

### Preparation of biopolymer solution for a healthy skin base

The skin base compositions were formulated using agarose (low EEO grade) and a pink dye paste. To prepare the compositions, agarose powder was dispersed in a small amount of cold deionised water, comprising 10% of the total water content. Once dispersed, the remaining deionised water was added at a high temperature (> 80 °C), resulting in a 2% w/w agarose solution. The mixture was stirred and heated using a heated magnetic stirrer set to approximately 700 rpm until complete hydration and a uniform solution were achieved at around 85 °C. To achieve opacity, an appropriate quantity of pink dye paste was gently introduced into the solution. For different skin tones, varying amounts and colours of dye were added. This yielded the desired healthy skin base material.

### Preparation of artificial slough

To create the artificial sloughy tissue, a suitable amount of custard powder was measured to achieve a 15% w/w solution. The measured powder was then added to approximately 25 ml of cold deionised water (per model). The solution was heated to ensure complete dissolution of the custard powder and left to thicken. Once thickened, the mixture was cooled to 4 °C. Before using the gelled custard as slough in the wound model, it was stirred to obtain a granular texture.

### Preparation of artificial eschar solution

The necrotic eschar material was formulated using agarose (low EEO grade) and black dye paste. Agarose powder was dispersed in a small portion of cold deionised water, constituting 10% of the total water content. Once dispersed, the remaining deionised water was added at a high temperature (> 80 °C) to create a 2% w/w agarose solution. The mixture was stirred and heated using a magnetic stirrer, set to approximately 700 rpm, until complete hydration and a uniform solution was achieved at around 85 °C. To achieve opacity, an appropriate amount of black dye paste was gently incorporated into the solution.

### Wound Model Assembly

The hot agarose skin base solution (as prepared in the “Preparation of biopolymer solution for a healthy skin base” section) was poured into a 3D printed mould that contained the shape and dimensions of the desired wound. In a separate mould that contained the shape and dimensions of the required necrotic eschar, a hot black agarose solution (as prepared in the “Preparation of artificial eschar solution” section) was added. These were both then allowed to set at room temperature. Once fully set, the wound bed was gently removed from the mould, and any imperfections were trimmed away around the edges using a scalpel. A drop of red food colouring was added to the wound cavity and carefully spread evenly throughout the cavity. A second drop of red food colouring was added into the centre of the wound cavity to make a darker red colour at the centre for the simulation of healthy granulation tissue, then left for 5 min. The artificial slough was then added to fill the cavity using a spatula, until flush with the healthy skin region. The gelled agarose eschar was removed from the mould, placed on top of the artificial slough, and gently pressed into the slough until flush with the healthy skin region. The top of the eschar was then cauterised from the edge towards the centre using a blow torch until a thin film was visible on the surface and the edges were bound to the healthy skin region.

### Mechanical characterisation of wound model components

To conduct rheological analysis of the skin base and sloughy tissue, small deformation oscillatory measurements were performed using a Malvern Gemini Rheometer (Malvern Instruments, UK) equipped with a 40-mm parallel plate geometry and Peltier plate thermal control. The elasticity of the materials was determined by conducting amplitude sweeps, which measured changes in the elastic modulus (G′) as a function of strain. Frequency sweeps were also performed to produce mechanical spectra of the samples. The elastic modulus (G′) and the viscous modulus (G″) were measured as a function of angular frequency (0.1–100 rad s^−1^ angular frequency). Measurements were taken at 20 °C and performed at 0.5% strain (strain amplitude chosen was within the linear viscoelastic region of the sample determined from amplitude sweeps). Viscosity measurements of the artificial slough were also taken as a function of shear rate to analyse any changes in viscosity or flow behaviour. Samples were exposed to increasing shear rates from 1 to 1000 s^−1^ using a 40-mm cone and plate geometry at a constant temperature of 20 °C.

A texture analyser (Stable Micro Systems, UK) was employed to assess the mechanical properties of artificial eschar using tensile testing. Prior to use, the texture analyser was calibrated according to the manufacturer’s instructions. Artificial eschar gels were prepared in a dog-bone geometry by using a custom 3D printed cutter and loaded between tensile clamps for tensile testing. Tests were performed at room temperature using a test speed of 2 mm/s. Peak stress was identified as the maximum stress achieved before breaking using stress–strain curves. Tensile strength for each repetition was calculated by dividing the maximal tensile force by the stress area of the artificial eschar gel (33.75 mm^2^). Young’s modulus was extracted from the linear region of the stress–strain curve.

## Results

**Fig. 1:**
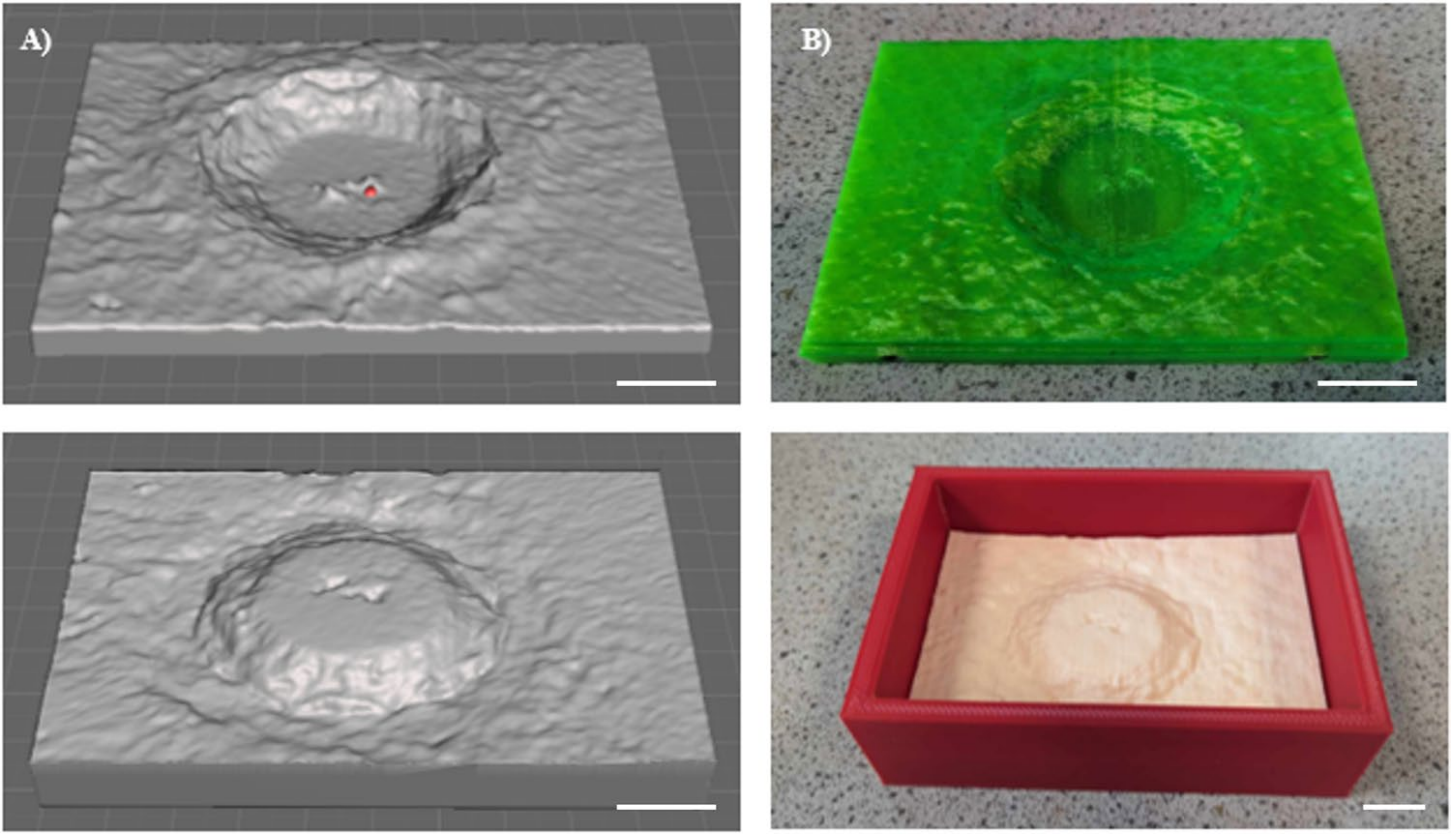
Wound mould designs for the fabrication of eschar (upper) and wound base (lower) in **A** STL file format and **B** 3D printed format (scale bar = 2 cm)

The 3D printed moulds prepared using PLA had a mottled texture (Fig. 1) when complete, which was subsequently imprinted on the healthy skin base surface, giving the material further resemblance to human skin.

**Fig. 2:**
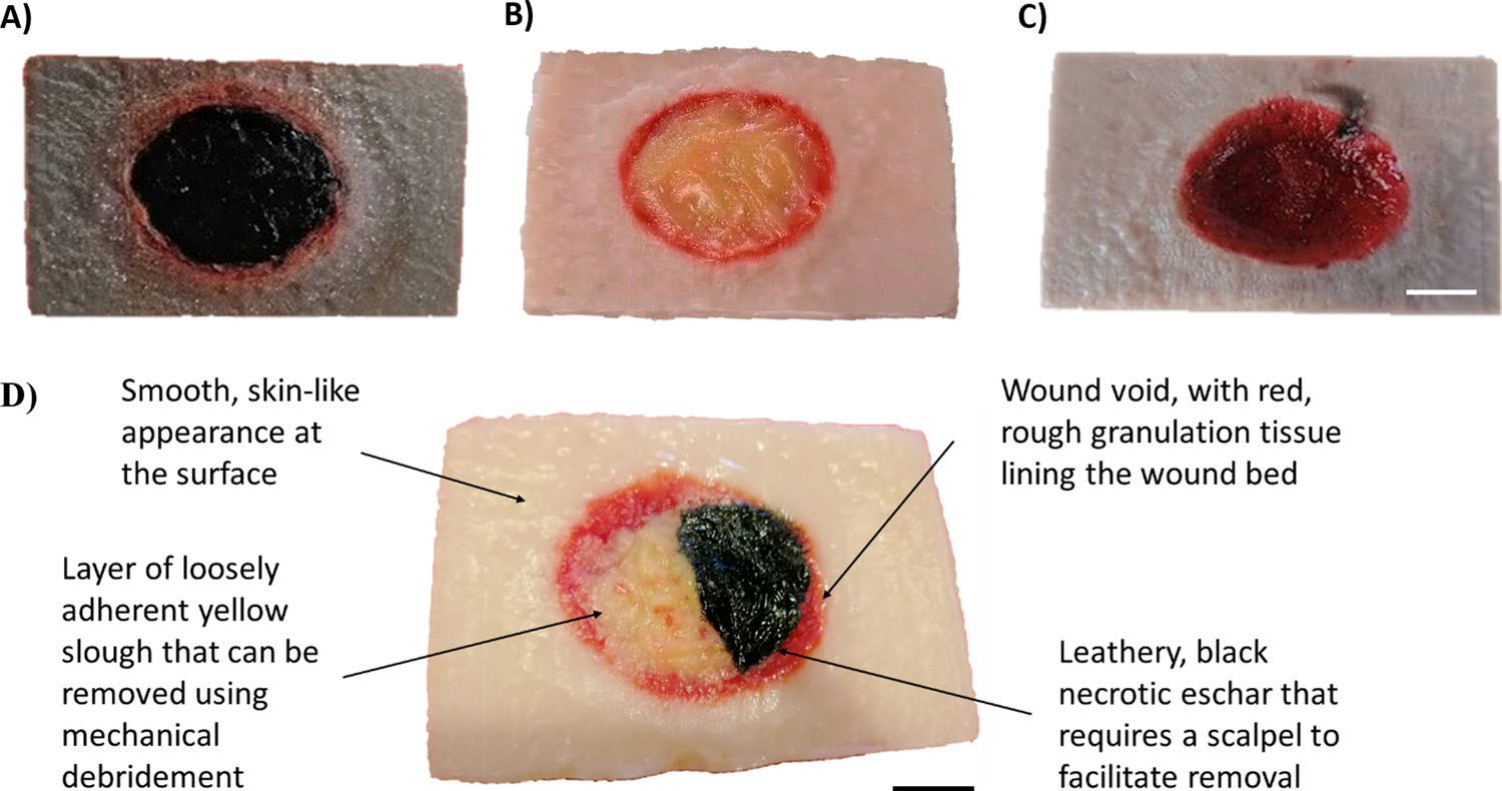
Images of prepared wound models highlighting the different layers and skin tones. **A** Necrotic eschar, **B** sloughy layer underneath the eschar, and **C** lower granulation tissue visible following successful debridement and **D** visual representation of all three layers (scale bar = 2 cm)

The wound model was assembled to have three main layers: the red granulation tissue on the healthy skin base (Fig. 2C), yellow sloughy tissue (Fig. 2B), and a necrotic eschar (Fig. 2A). This provided an appearance across all the layers (Fig. 2D) that was similar to what is observed in real chronic wounds that require debridement.

**Fig. 3:**
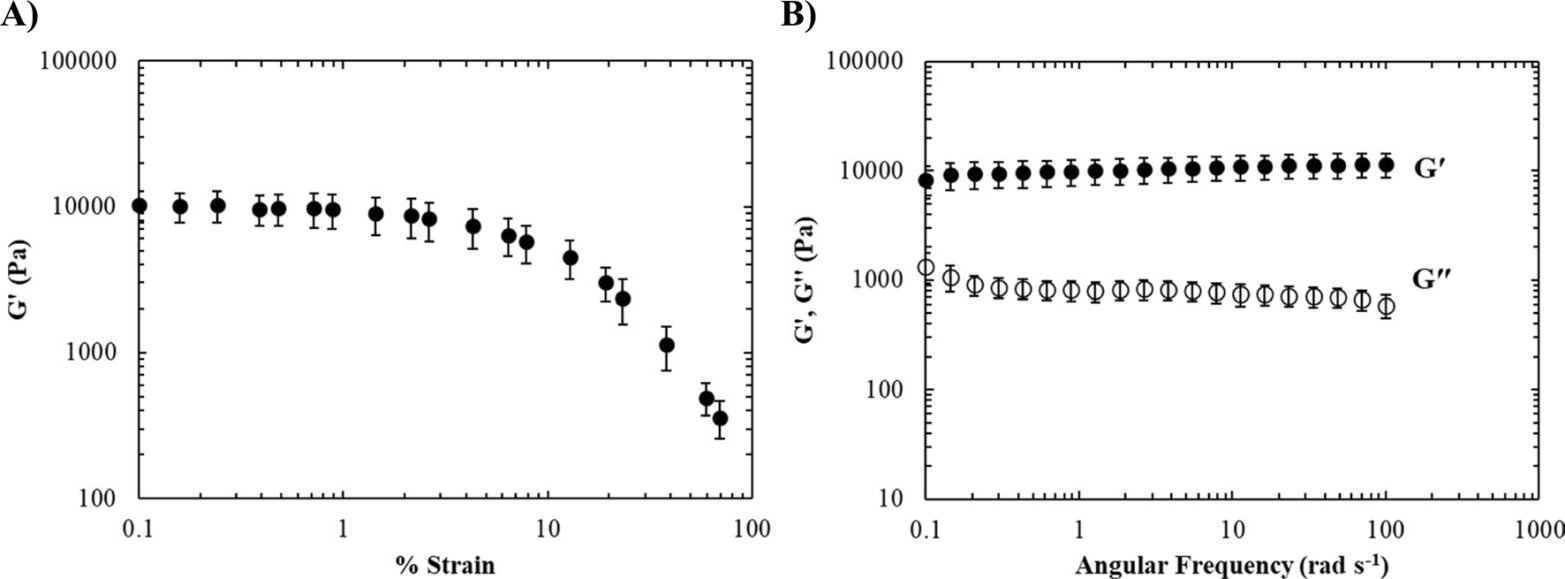
Mechanical properties of the healthy skin base. **A** Amplitude sweep showing the LVR and **B** frequency sweep showing mechanical spectra (mean values ± SD *n* = 3)

To ensure that the physical properties of the model were similar to those of a real chronic wound, various material testing measurements were performed. The rheological properties of the healthy skin base were assessed with an amplitude sweep, which shows the extent of the linear viscoelastic region (LVR) and the strain where failure begins to occur (Fig. 3A). The strength of the materials was measured using a frequency sweep, which revealed strong gel-like properties with G′ ~ 10,000 Pa and G″ more than an order of magnitude lower across all frequencies tested (Fig. 3B).

**Fig. 4:**
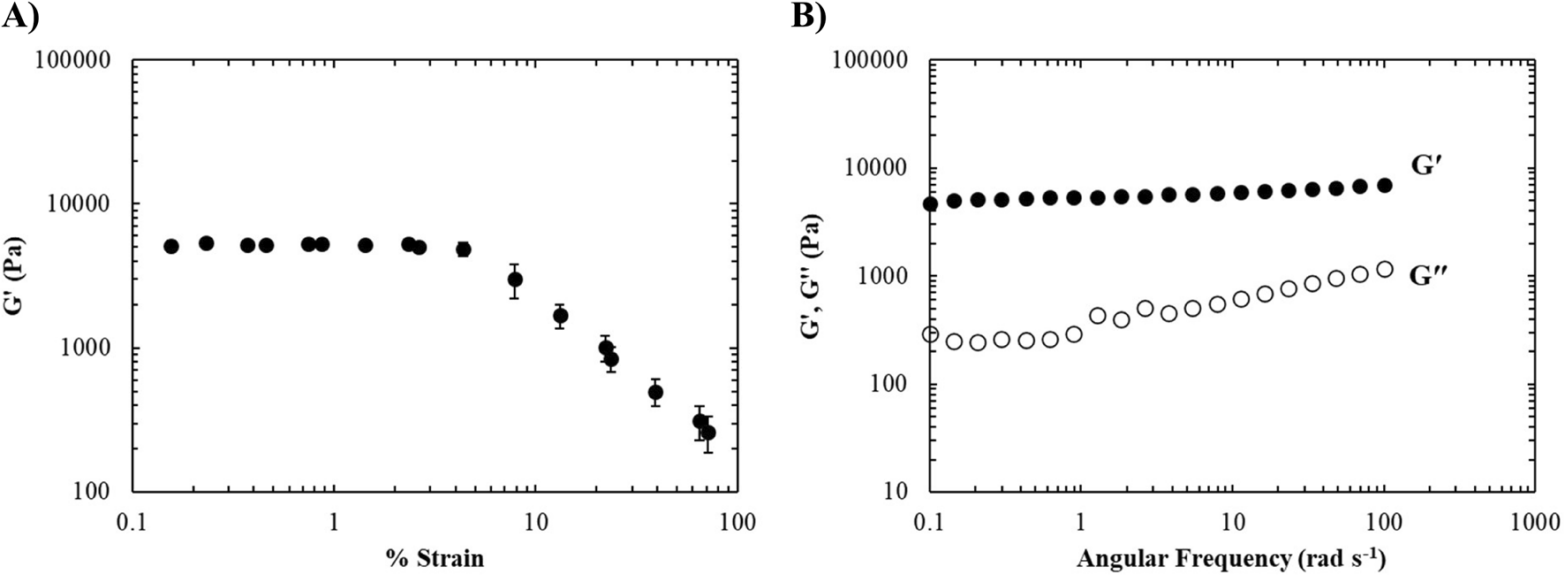
Mechanical properties of the artificial sloughy tissue. **A** Amplitude sweep showing the LVR and **B** frequency sweep showing mechanical spectra (mean values ± SD *n* = 3)

**Fig. 5:**
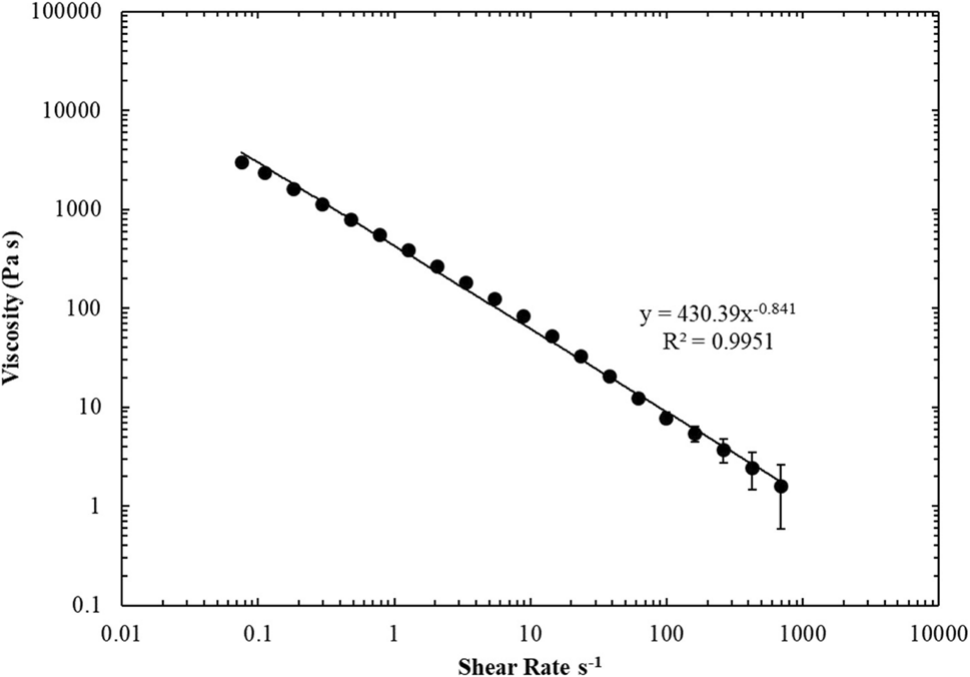
Viscosity vs. shear rate showing shear thinning behaviour of the artificial slough (mean values ± SD *n* = 3)

The sloughy tissue was shown to have a larger elastic region than the healthy skin base (Fig. 4A) and a mechanical spectrum that indicated gel-like properties that were somewhat weaker than the healthy skin base indicated by a gradual increase in G″ at higher frequencies (Fig. 4B). Steady shear experiments on the slough were also performed, which clearly indicated shear thinning flow behaviour, under shear conditions that the material would encounter during debridement (Fig. 5).

**Fig. 6:**
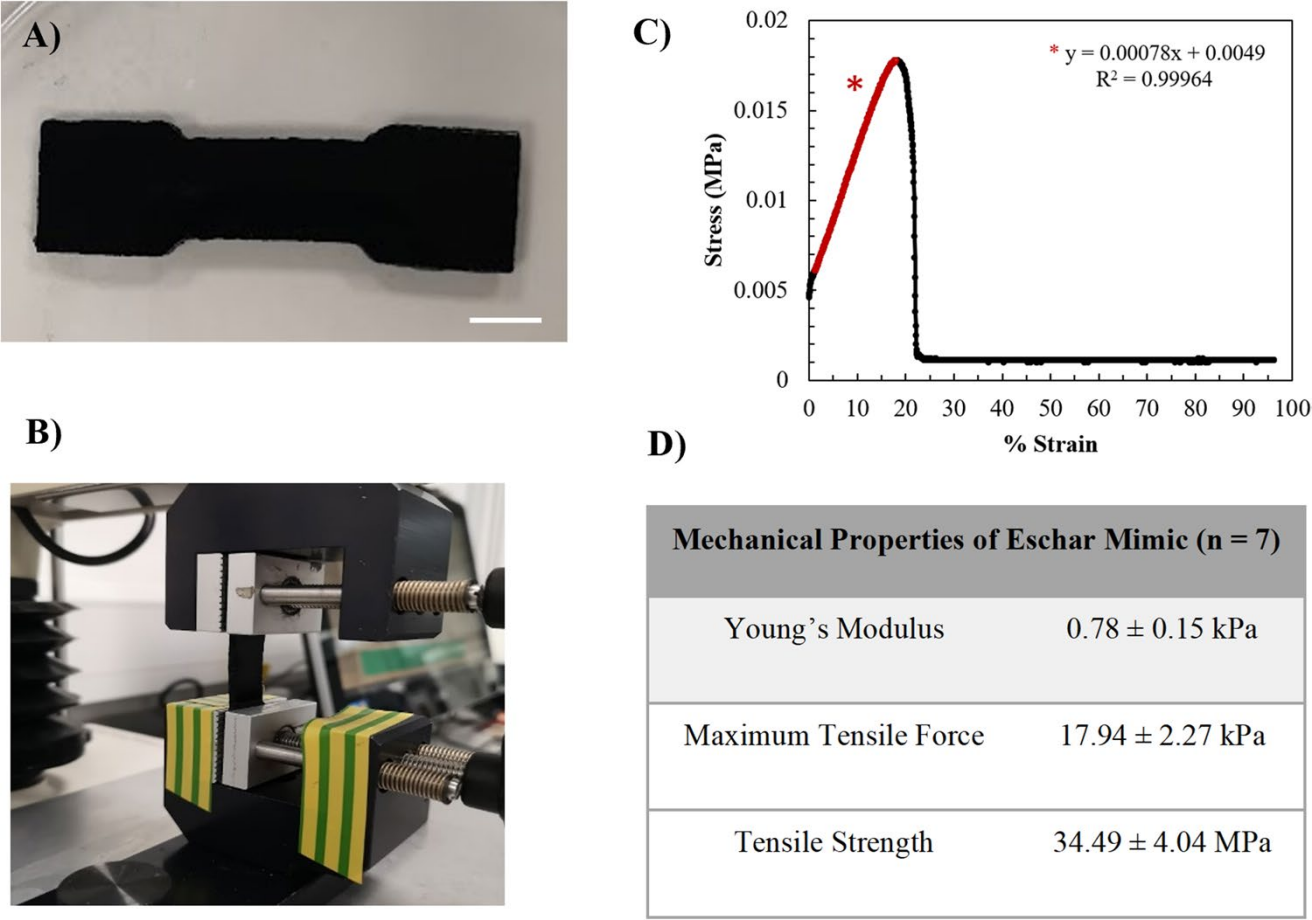
Mechanical properties of simulated necrotic eschar. **A** Dog bone–shaped artificial eschar gel (scale bar = 1 cm) **B** loaded between two clamps of a texture analyser. **C** Stress–strain curves demonstrating the brittle nature of artificial eschar and **D** key mechanical properties including Young’s modulus, maximum tensile force, and tensile strength (mean values ± SD *n* = 7)

To quantify the mechanical properties of the eschar layer, a texture analyser was used to perform elongation measurements to generate force deformation curves. Dog bone–shaped eschar material (Fig. 6A) was secured with clamps on the texture analyser (Fig. 6B), strain was measured as a function of stress, and mechanical properties were then calculated from the resulting force deformation curve, which showed characteristic brittle fracture behaviour (Fig. 6C). The material had an average Young’s modulus of 0.78 kPa and a tensile strength of 34.49 MPa (Fig. 6D).

**Fig. 7:**
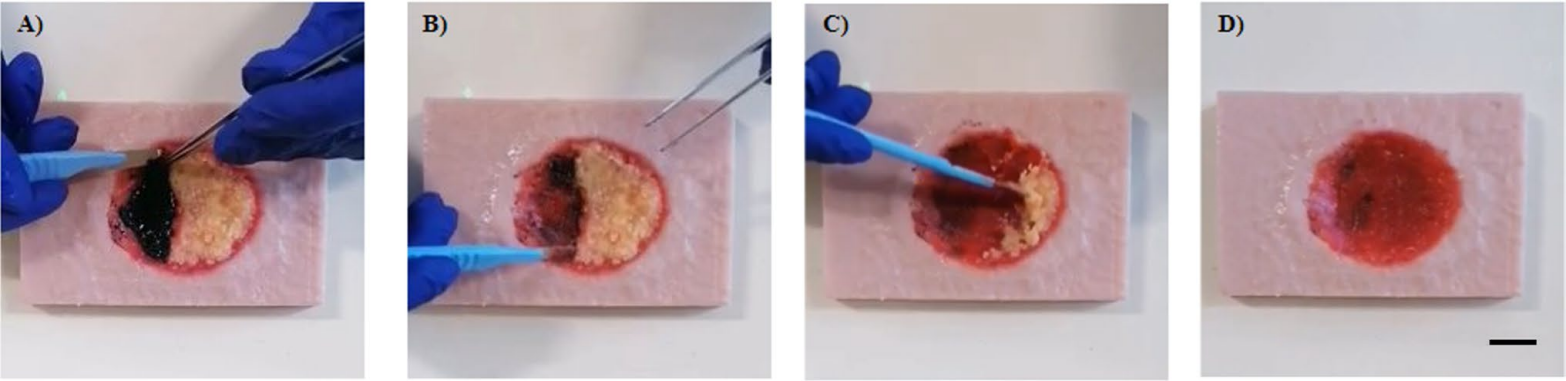
Model stills during the debridement process **A** prior to debridement, **B** following removal of eschar, **C** during debridement of slough, and **D** after treatment (video in supplementary material) (scale bar = 2 cm)

Demonstration of the debridement process was then performed on an assembled model in the same manner as would be performed by a trainee showing the similarities with the debridement of a real chronic wound (Fig. 7) (see supplementary video (S1)).

## Discussion

Developing an anatomically realistic model that replicates the appearance and textures of real-life chronic wounds has the potential to improve debridement training, subsequently resulting in more confident and capable practitioners. Moreover, the ease of manufacturing the model provides the capability to increase training opportunities for health care professionals, which is urgently needed due to an ageing population and the inevitable increases in the number of patients with chronic wounds. To this end, a model has been manufactured and developed using natural, sustainable materials without the incorporation of animal-based products. Each layered component within this interactive model carefully recapitulates the visual and physical characteristics found in chronic wounds, providing realistic tactile and haptic feedback and creating a platform conducive to training a range of debridement techniques. In preparation, a 3D printed mould was used to cast the base of the skin mimic, within which the wound void is situated. The advantage of using a 3D printed mould is that it allows for the production of identical moulds for reproducible batches on a large scale and the potential opportunity to create bespoke wound shapes and surface textures and generate more complex wound models that may feature undermining or tunnelling and other anatomical features frequently observed during debridement. The base healthy skin layer was made using agarose, which was tuned by controlling the concentration to form a strong and firm hydrogel with similar mechanical properties to that of ageing human skin to provide a realistic feel when debriding the model. In addition, the agarose can be dyed when preparing the precursor agarose solution to match a range of different skin colours and tones, providing further realism. The wound void was dyed red and appears to look like rough granulation tissue with some light bleeding lining the wound bed. Furthermore, the dye has the ability to move into small porosities in the agarose gel at the edges of the wound void, appearing like small blood vessels protruding into the healthy skin. Above the granulation tissue is a layer of loosely adherent viscoelastic slough prepared from yellow custard powder that can be removed using mechanical debridement. The uppermost layer of the wound region exhibits leathery black necrotic eschar prepared from concentrated agarose hydrogel that was dyed black. The hydrogel was dehydrated during the building of the wound layers to achieve the leathery appearance and was loosely integrated into the healthy skin, requiring a scalpel to facilitate removal, as is often the case in real-life wounds. To ensure the model has a realistic appearance along with similar tactile behaviour, mechanical properties of the model layers were matched to be similar to those observed in human tissue. The skin base was shown to have a similar elastic behaviour to full-thickness adult healthy skin [18] with a linear viscoelastic region (LVR) lying between 0.1 and 1% strain (Fig. 3A). The frequency sweeps of the skin base (Fig. 3B) showed strong gel behaviour with G′ > G″ across all frequencies measured with values in the same order of magnitude of full thickness healthy skin.

The mechanical properties of human sloughy tissue have not been reported in the literature to date. However, qualitative terminology is routinely used by clinicians to describe and categorise the physical properties. Accordingly, we developed the artificial slough by presenting various formulations to specialist tissue viability nurses for evaluation of the physical properties of those found in real-life patients. Following this consultation, the rheological properties of the selected slough formulation were measured. Strain sweeps conducted on the sloughy tissue (Fig. 4A) revealed an extension of the LVR up to approximately 10% strain, indicating the elastic nature of the slough. Additionally, the frequency sweep data demonstrated a solid-like behaviour, with the storage modulus (G′) consistently higher than the loss modulus (G″) across all measured frequencies. However, at higher frequencies, the loss modulus (G″) showed an increase, suggesting a breakdown of polymer interactions (Fig. 4B). This observation suggests that the slough exhibits solid-like characteristics at lower frequencies while transitioning towards a more viscous behaviour at higher frequencies. This unique combination of solid-like behaviour at low frequencies and increased viscosity at higher frequencies allows the slough to remain stable within the wound when at rest. However, during debridement, the slough exhibits enhanced viscous behaviour, resembling that of native sloughy tissue. Therefore, when subjected to increasing shear forces, slough undergoes a reduction in viscosity, allowing it to flow more easily. This property is generally beneficial during wound debridement as it enables easier removal and manipulation of slough, as is the case in the developed model. While the rheological properties of human sloughy tissue have not previously been measured, it has been reported that more viscous slough that is clinically associated with inflamed and heavily infected wounds (which are of the kind we aimed to replicate) is likened to that of other bodily fluids such as respiratory mucus with viscosity values as high as 10^3^ Pa s [19]. Moreover, mucus also exhibits shear thinning behaviour, which is also apparent in the qualitative analysis of native sloughy tissue. Indeed, the slope of the viscosity vs. shear rate for mucus is commonly within the range of − 1 to − 0.5, with an average of − 0.85 [20]. The artificial slough also demonstrated shear thinning flow behaviour with a slope of − 0.84 (Fig. 5).

The mechanical properties of artificial eschar were measured by fabricating artificial eschar gels in a dog-bone geometry using a 3D printed template. The gels were then loaded onto a texture analyser equipped with tensile clamps for testing, which collected stress–strain data (Fig. 6A–C). A representative stress–strain curve is displayed in Fig. 6C, which initially shows a linear relationship between stress and strain, followed by a rapid decrease in stress after reaching the peak stress (maximum tensile force), with no clear plastic deformation region observed—a typical response of brittle materials. Real eschar has been reported in the literature as also being brittle in nature [21], making concentrated agarose-based artificial eschar a promising candidate as a necrotic eschar mimic for bio-inspired wound models.

The slope of the curve was determined to reveal Young’s modulus of the material, which is indicative of material stiffness (Fig. 6C). Artificial eschar exhibited an average stiffness of 0.78 ± 0.15 kPa. Tensile strength was also determined using the maximum tensile force reached during the experiment (17.94 ± 2.27 kPa) and dimensions of the gel stress area (33.75 mm^2^). For artificial eschar, the tensile strength was determined to be 34.49 ± 4.04 MPa. While these findings indicate the artificial eschar was relatively low in stiffness and strength, the mechanism of fracture and realistic organoleptic properties demonstrates to trainees how eschar will likely behave during debridement. Furthermore, the tuneable nature of the biopolymers used to make the eschar provides scope to further optimise the mechanical properties if required.

Once the model was fully assembled, it was clear that there was sufficient integration of the eschar and slough with the wound base that required the debrider to use similar forces that would normally be required for debriding a real wound (S1). Moreover, the realistically matched mechanical properties of each layer provide realistic haptic feedback for the trainee helping to equip the clinician with the required skill in a more biologically relevant manner when compared with using fruit peel or pig’s feet. An additional benefit of the developed model is that it is composed of sustainable biopolymers. This offers a cost-effective and scalable alternative to current training methods. Biopolymers derived from renewable sources are not only environmentally friendly but also cost-effective due to their abundance and ease of production. Unlike the limited availability and perishable nature of pigs’ feet and fruit peel, sustainable biopolymers can be manufactured and purchased in large quantities at a lower cost, ensuring scalability for widespread training use. Additionally, biopolymers provide consistency in texture, properties, and durability, offering a standardised training experience that closely mimics real-life scenarios without the ethical concerns or variability associated with animal-derived materials. This approach not only reduces training costs but also promotes sustainability and innovation in medical education. Use of this model will therefore overcome some of the current issues associated with debridement training and result in a better trained and more confident debridement clinicians with improved clinical outcomes for patients.

## Conclusion

Here, an anatomically realistic, disposable, chronic wound model has been developed utilising sustainable natural materials that are biodegradable and non-toxic. This model has the potential to serve as an effective tool for trainee clinicians to practice sharp debridement safely. Each layer of the model was designed to replicate the visual and physical characteristics observed in chronic wounds, ensuring a realistic tactile experience and providing accurate haptic feedback. By incorporating these features, the model creates an ideal platform for training a diverse range of debridement techniques.

Currently, there is a distinct lack of documentation of the mechanical and viscoelastic properties of native wound tissue, relying predominantly on clinical observations. Conducting a thorough evaluation of each respective ‘layer’ of a wound and obtaining a physiological range of mechanical and viscoelastic behaviours for each component hold the potential to significantly advance the visual and tactile features of our model. Combined with design flexibility (as provided through computer-aided design and 3D printing of custom moulds), the model could also be further enhanced to better suit various debridement scenarios, reflecting and providing debridement training for the extensive range of wound presentations observed in real clinical scenarios.

## Supplementary Information

Below is the link to the electronic supplementary material.

Supplementary file1 (MP4 152606 KB)

## Data Availability

The authors declare that the data supporting the findings of this study are available within the paper, its supplementary information files,
